# Crystal Structures of Tcl1 Family Oncoproteins and Their Conserved Surface Features

**DOI:** 10.1100/tsw.2002.826

**Published:** 2002-07-04

**Authors:** John M. Petock, Ivan Y. Torshin, Yuan-Fang Wang, Garrett C. Du Bois, Carlo M. Croce, Robert W. Harrison, Irene T. Weber

**Affiliations:** Department of Biology, Georgia State University, Atlanta, GA, USA; Department of Computer Science, Georgia State University, Atlanta, GA, USA; Department of Microbiology and Immunology, Kimmel Cancer Center, Thomas Jefferson University, Philadelphia, PA, USA

**Keywords:** leukemia, oncoprotein, Tcl1, Akt/protein kinase B, X-ray crystallography

## Abstract

Members of the TCL1 family of oncogenes are abnormally expressed in mature T-cell leukemias and B-cell lymphomas. The proteins are involved in the coactivation of protein kinase B (Akt/PKB), a key intracellular kinase. The sequences and crystal structures of three Tcl1 proteins were analyzed in order to understand their interactions with Akt/PKB and the implications for lymphocyte malignancies. Tcl1 proteins are ~15 kD and share 25—80% amino acid sequence identity. The tertiary structures of mouse Tcl1, human Tcl1, and Mtcp1 are very similar. Analysis of the structures revealed conserved semi-planar surfaces that have characteristics of surfaces involved in protein-protein interactions. The Tcl1 proteins show differences in surface charge distribution and oligomeric state suggesting that they do not interact in the same way with Akt/PKB and other cellular protein(s).

## ROLE OF TCL1 IN LYMPHOCYTE MALIGNANCY

The TCL1 family of oncogenes are abnormally expressed in mature T-cell leukemias and B-cell lymphomas[1,2]. This gene family includes human TCL1 (T-Cell Leukemia/lymphoma 1), TCL1b, and MTCP1 (Mature T-Cell Proliferation 1). Both TCL1 and TCL1b genes are found at

```
Structure    --b--       ---b---  --b--   -----b----- -----b----
hTcl1   1 MAECPTLGEAVTDHPDRLWAWEKFVYLDEKQHAWLPLTIEIKDRLQLRVLLRRE
mTcl1   1 MATQRAHRAETPAHPNRLWIWEKHVYLDEFRRSWLPVVIKSNEKFQ  VILRQE
hMtcp1  1     GSAGEDVGAPPDHLWVHQEGIYRDEYQRTWVAVVEEETSFLR  ARVQQI
                            ---b---  --b-   -----b---- ----b---
Consensus               P rLW     vY DE  r Wv vv       q  V lr e

Structure          ---a---    ---b--   --b--  -----b-----  -----b----
hTcl1   55 DVVLGRPMTPTQIGPSLLPIMWQLYPDGRYRSSDSSFWRLVYHIKIDGVEDMLLELLPDD
mTcl1   53 DVTLGEAMSPSQIVPYELPLMWQLYPKDRYRSCDSMYWQILYHIKFRDVEDMLLELIDSESNDE
hMtcp1  49 QVPLGDAARPSHLLTSQLPLMWQLYPEERYMDNNSRLWQIQHHLMVRGVQELLLKLLPDD
                   ---a---    ---b--- ---b--  -----b-----  ----b----
Consensus   V lg      q   s LP mWQLYP  rYr  dS fW I  H     ve llL l p
```

**FIGURE 1.** Sequence alignment of Tcl1 and Mtcp1 proteins. Sequences are shown for human Tcl1 (hTcl1), mouse Tcl1 (mTcl1), and human Mtcp1 (hMtcp1). The elements of secondary structure are shown for human Tcl1 and Mtcp1 with "a" indicating the short helix and "b" for beta strand. The conserved serine (Ser89 in human Tcl1) is shown in bold.

the human TCL1 locus. TCL1 genes are expressed in embryonic tissues and immature B- and T-cells. Mature T-cells do not normally express TCL1 genes. Abnormal expression in mature lymphocytes is induced by chromosomal rearrangements that position the regulatory elements of T-cell receptor genes next to the TCL1 locus[2]. The TCL1 locus is activated in the majority of cases of T-cell prolymphocytic leukemia and T-cell chronic lymphocytic leukemia. Rearrangements involving MTCP1 are less commonly associated with mature T-cell leukemia. The oncogenic nature of TCL1 and MTCP1 has been confirmed by analysis of transgenic mouse models[3,4].

Members of the TCL1 family encode for ~15-kD proteins that share 25–80% amino acid sequence identity. The sequences of human Tcl1, mouse Tcl1, and Mtcp1 proteins are shown in Fig. 1. The normal physiological role of Tcl1 proteins is not well defined. Functional analysis revealed that Tcl1 proteins coactivate protein kinase B (Akt/PKB)[5,6]. No other protein is known to interact with Tcl1 proteins. Akt/PKB has a key role in diverse cellular processes including glucose metabolism, transcription, cell growth, survival, and migration[7]. Therefore, interactions between Tcl1 and Akt/PKB are likely to be important for the functioning of the whole cell. Akt/PKB is a serine/threonine kinase that comprises three functional domains: an N-terminal pleckstrin homology (PH) domain, a catalytic (kinase) domain, and a C-terminal regulatory domain. A Tcl1 dimer is proposed to bind to the PH domain forming an oligomeric complex with Akt/PKB, followed by Akt/PKB activation by other kinases. The activated Akt/PKB can phosphorylate many proteins involved in cell proliferation and survival. Therefore, abnormal coactivation of Akt/PKB by Tcl1 in mature lymphocytes can lead to cancer.

## ANALYSIS OF TCL1 AND MTCP1 PROTEIN STRUCTURES

The crystal structures were determined for the recombinant proteins human Tcl1 (PDB identifier 1JSG)[8], mouse Tcl1 (1JNP)[9], and human Mtcp1 (1A1X)[10]. These structures were determined at 2.0–2.5 Å resolution. The refinement parameters and statistics are summarized in Table 1. Whereas human Tcl1 and Mtcp1 were solved as monomers in the $I222$ and $P6_222$ space groups, respectively, the mouse Tcl1 crystal structure was solved as a dimer in the $C2$ space

**TABLE 1**
**Crystallographic Statistics for Human Tcl1, Mouse Tcl1, and Human Mtcp1 Structures**

| | Human Tcl1 | Mouse Tcl1 | Human Mtcp1 |
|---|---|---|---|
| Space group | $I_{222}$ | $C_1 2_1$ | $P6_{222}$ |
| Unit cell | 38.9, 82.8, 117.7 | 89.3, 115.9, 37.9 | 62.7, 62.7, 86.0 |
| | 90.0°, 90.0°, 90.0° | 90.0°, 115.2°, 90.0° | 90.0°, 90.0°, 120° |
| *R* merge | 0.09 | 0.06 | 0.05 |
| Resolution | 2.5 Å | 2.5 Å | 2.0 Å |
| *R* factor | 19% | 23% | 21% |
| *R* free | 26% | 24% | 25% |
| Bond length error | 0.006 | 0.02 | 0.007 |
| Angle error | 1.3 | 2.1 | 1.4 |

**TABLE 2**
**Structural Similarity for Mouse Tcl1, Human Tcl1, and Human Mtcp1**

| | Mouse Tcl1A* | Mouse Tcl1B | Human Tcl1 | Human Mtcp1 | Internal Symmetry |
|---|---|---|---|---|---|
| Mouse Tcl1A | - | 0.9 (100%) | 0.6 (50%) | 1.6 (36%) | 1.3 (12%) |
| Mouse Tcl1B | 0.9 (100%) | - | 0.6 (50%) | 1.5 (36%) | 1.1 (12%) |
| Human Tcl1 | 0.6 (50%) | 0.6 (50%) | - | 1.5 (41%) | 1.3 (13%) |
| Human Mtcp1 | 1.6 (36%) | 1.5 (36%) | 1.5 (41%) | - | 1.7 (13%) |

*Note*: The crystal structure of mouse Tcl1 comprises the two subunits Tcl1A and Tcl1B.

* Root mean square (RMS) differences are given in Ångstroms with the percentage amino acid sequence identity in parentheses.

group. The three Tcl1 proteins show different possible subunit–subunit interfaces in the crystals. In solution, Mtcp1 is likely to be a monomer, while human Tcl1 can form dimers[11]. Moreover, mouse Tcl1 may form trimers in solution[6]. Therefore, the oligomeric state and subunit interface may differ among these proteins.

The crystal structures of mouse Tcl1, human Tcl1, and Mtcp1 have very similar tertiary structures as shown in Fig. 2A. However, no other similar structures were found in the Protein Data Bank[12]. The sequences and structures of Tcl1 proteins are compared in Table 2. Human and mouse Tcl1 structures have a root mean square (RMS) difference of 0.6 Å for 100 superimposed Cα atoms and share 50% sequence identity, while human Tcl1 and Mtcp1 show 1.5 Å RMS difference for 100 Cα atoms and 41% sequence identity[10]. The structures of mouse Tcl1 and human Mtcp1 have 1.6 Å RMS difference for 100 aligned Cα atoms. The proteins share an 8-stranded beta barrel structure of unique topology. The beta barrel structure has a long surface loop with a short helical region that separates two 4-stranded pseudo-symmetric "half-barrel" structures (Fig. 2B). The three protein structures have high internal symmetry for the "half-barrel" structure with RMS differences ranging from 1.1–1.7 Å, despite the lack of significant sequence identity between the halves (12–13%).

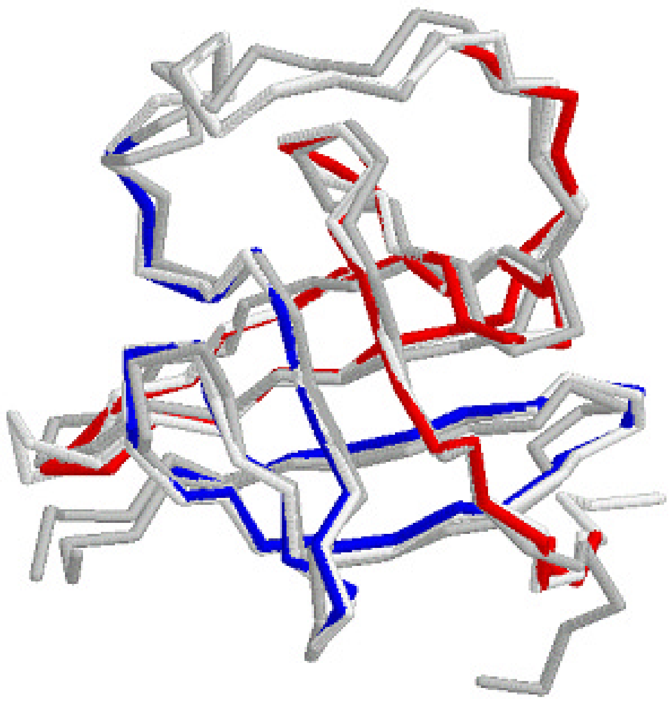

**FIGURE 2a.**

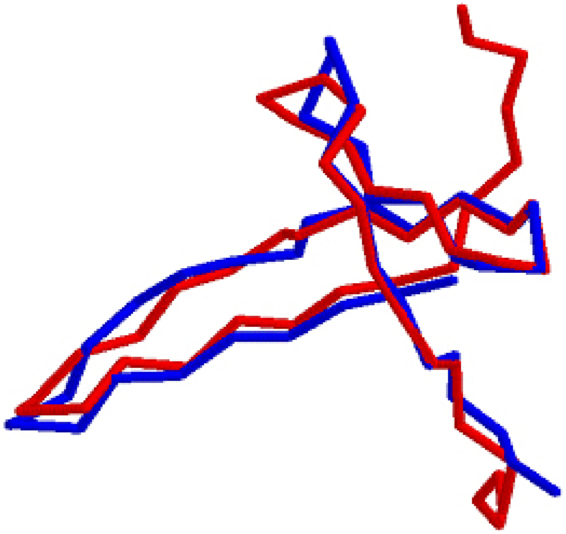

**FIGURE 2b.**

**FIGURE 2.** Superposition of alpha carbon backbone atoms of the Tcl1 family members: mouse Tcl1 (red for residues 8-57, blue for 67-108), human Tcl1 (white), and human Mtcp1 (gray). The red/blue color scheme is shown to emphasize the half-barrel structure shown in (B). Two tandem halves of mouse Tcl1 structure superimposed to show the internal symmetry (B). Residues 8-57 (red) and 67-108 (blue) are included.

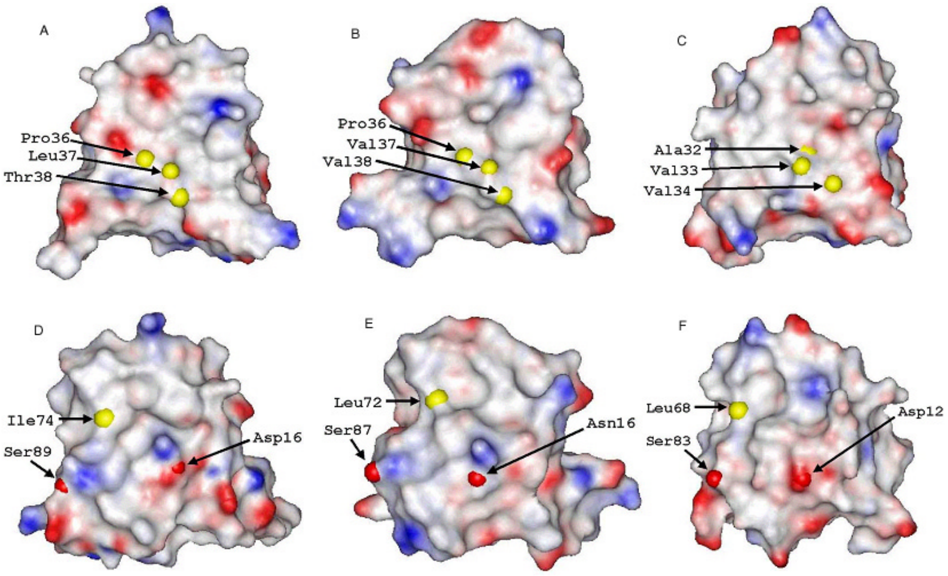

**FIGURE 3.** The largest conserved semi-planar surfaces of the Tcl1 protein members is shown with the surface charge distribution for: (A) human Tcl1, (B) mouse Tcl1, and (C) human Mtcp1. Blue indicates positive charge distribution and red indicates negative. The human Tcl1 residues Pro36, Leu37, and Thr38 found to be important for dimer formation are shown and equivalent residues are indicated in the other proteins. The Tcl1 surfaces proposed to interact with Akt/PKB are shown for: (D) human Tcl1, (E) mouse Tcl1, and (F) human Mtcp1. This surface is located opposite to the dimer surface shown in views A-C. The conserved serine is indicated. Human Tcl1 Asp16 and Ile74 found to be important for interactions with Akt are shown in D, and the equivalent residues in mouse Tcl1 and Mtcp1 are indicated in E and F. The surface pictures were generated with the program WebLab ViewerLite[16] using a probe radius of 2.5 Å.

## ANALYSIS OF TCL1 PROTEIN SURFACES

Human Tcl1 residues involved in homodimer formation and association with Akt/PKB have been identified recently[13]. Mutational analysis of human Tcl1 suggested that amino acids 35–37 are involved in dimer formation, while residues Asp16 and Ile74 are important for Tcl1-Akt/PKB interactions. It is not known whether the equivalent residues in mouse Tcl1 and human Mtcp1 have the same function. Presumably, additional Tcl1 residues are involved in these protein–protein interactions. Therefore, the molecular surfaces of Tcl1 and Mtcp1 structures were analyzed in relation to the human Tcl1 mutations. The Tcl1 proteins show three conserved nearly planar surfaces[10]. These large surfaces have characteristic features with many “knobs” and “clefts” similar to the interfaces observed in homodimers and other protein–protein complexes[14,15]. The Tcl1 mutations that interfere with dimer formation and interactions with Akt/PKB lie on two of these large surfaces.

The human Tcl1 residues 36–38 that were shown to be involved in dimer formation[13] lie on the largest of the conserved semi-planar surfaces of Tcl1 proteins. The proposed Tcl1 dimer-forming surface is roughly 500 Å$^2$ in size and is shown in Fig. 3A–C. However, the surface distribution of negative and positive charges is different in each of the Tcl1 proteins. We identified the surface accessible residues comprising this dimer interface in all three Tcl1 crystal structures (Table 3). These residues are *Glu22*, *Leu35*, *Leu37*, Thr38, Ile39, Glu40, Ile41, Lys42, Leu50, Arg52, Glu54, **Val56**, Val57, Arg60, Thr63, Gln66, Pro69, Ser70, Leu71, Ile100, Asp101, and **Val103** in human Tcl1 (identical residues in bold, similar residues in italics). Only 2 out of 22 surface residues are identical and 3 are similar in all three proteins (23% [5/22] conserved residues). Thus, the majority of residues forming this surface differ among the Tcl1 proteins.

**TABLE 3**
**Comparison of Surface Residues for Human Tcl1, Mouse Tcl1, and Human Mtcp1 Proteins**

| Akt-Interacting Surface | | | Dimer Forming Surface | | |
|---|---|---|---|---|---|
| Human Tcl1 | Mouse Tcl1 | Human Mtcp1 | Human Tcl1 | Mouse Tcl1 | Human Mtcp1 |
| *Asp16* | Asn16 | *Asp12* | *Glu22* | *Glu22* | *Gln18* |
| Arg17 | *Arg17* | *His13* | *Leu35* | Leu35 | *Val31* |
| **Trp19** | **Trp19** | **Trp15** | *Leu37* | *Val37* | *Val33* |
| Trp21 | Trp21 | His17 | Thr38 | Val38 | Val34 |
| **Glu29** | **Glu29** | **Glu25** | Ile39 | Ile39 | Glu35 |
| Lys30 | Phe30 | Tyr26 | Glu40 | Lys40 | Glu36 |
| Gln31 | Arg31 | Gln27 | Ile41 | Ser41 | Glu37 |
| Arg60 | Glu58 | Asp54 | Lys42 | Asn42 | Thr38 |
| Pro61 | Ala59 | Ala55 | Leu50 | Ile48 | Arg44 |
| Thr63 | Ser61 | Arg57 | Arg52 | Arg50 | Gln46 |
| **Pro64** | **Pro62** | **Pro58** | Glu54 | Glu52 | Ile48 |
| *Ile74* | *Leu72* | *Leu68* | **Val56** | **Val54** | **Val50** |
| **Met75** | **Met73** | **Met69** | Val57 | Thr55 | Pro51 |
| **Gln77** | **Gln75** | **Gln71** | Arg60 | Glu58 | Asp54 |
| **Tyr79** | **Tyr77** | **Tyr73** | Thr63 | Ser61 | Arg57 |
| **Pro80** | **Pro78** | **Pro74** | Gln66 | Gln64 | His60 |
| Asp81 | Lys79 | Glu75 | Pro69 | Pro67 | Thr63 |
| **Arg83** | **Arg81** | **Arg77** | *Ser70* | Tyr68 | Ser64 |
| Arg85 | Arg83 | Met79 | Leu71 | Glu69 | Gln65 |
| Ser87 | Ala85 | Asn81 | Ile100 | Phe98 | Val94 |
| Asp88 | *Asp86* | *Asn82* | Asp101 | Arg99 | Arg95 |
| **Ser89** | **Ser87** | **Ser83** | **Val103** | **Val101** | **Val97** |
| Ser90 | Met88 | Arg84 | | | |

The molecular surface of human Tcl1 that interacts with Akt/PKB can be identified from the two residues Asp16 and Ile74 that were shown to be important for Tcl1-Akt/PKB interactions[13]. The tentative Akt/PKB binding surface of Tcl1 corresponds to another conserved semi-planar region in the structures of human Tcl1, mouse Tcl1, and human Mtcp1, as shown in Fig. 3D–F. The surface accessible residues on the proposed Akt/PKB binding surface of human Tcl1 are *Asp16*, *Arg17*, **Trp19**, Trp21, **Glu29**, Lys30, Gln31, Arg60, Pro61, Thr63, **Pro64**, *Ile74*, **Met75**, **Gln77**, **Tyr79**, **Pro80**, Asp81, **Arg83**, Arg85, Ser87, *Asp88*, **Ser89,** and Ser90 (identical residues in bold, similar residues in italics). These 23 residues are likely to be important for interacting with Akt/PKB in the three Tcl1 proteins. Out of 23 residues forming the surface, 9 are identical and 4 are similar residues, suggesting a higher degree of conservation than in the putative dimer interface described above (52% [13/23] compared to 23%). Interestingly, the residues Asp16 and Ile74 shown to be important for interactions with Akt/PKB are not identical in all three proteins. Like the largest semi-planar surface of Tcl1 proteins, the charge distributions of the interacting surfaces differ among the three proteins. This suggests that different Tcl1 proteins do not bind in an identical way to Akt/PKB. Indeed, a difference in affinity for Akt/PKB was observed in studies with Tcl1, Tcl1b, and Mtcp1[5,6]. The activation of

Akt/PKB by interaction with Tcl1 was tenfold higher than that of Mtcp1. In addition, the three isoforms of Akt/PKB differ in their specificity for Tcl1 oncoproteins; isozyme Akt3/PKB-γ is most specific for Tcl1[18].

The potential phosphorylation sites differ for Tcl1 proteins[7]. Mouse Tcl1 shows a potential CK2 phosphorylation site at residues 55–58 on the dimer interface and a potential site for tyrosine kinase phosphorylation at residues 83–89 on the putative Akt/PKB interacting surface. No known phosphorylation sites were found in the Mtcp1 sequence. These differences in the location and the types of potential phosphorylation sites in relation to the molecular surfaces suggest that the three Tcl1 proteins interact with different cellular kinases.

## IMPLICATIONS FOR TCL1 INTERACTIONS WITH AKT/PKB

The overall coactivation of Akt/PKB by Tcl1 appears to be a complex process[5,6,13,19,20,21]. Initially, a growth factor signal activates cell surface receptors and causes phosphoinositide 3-kinase (PI3K) to produce phosphoinositides. These products bind to the PH domain of Akt/PKB for its recruitment to the plasma membrane. Next, a Tcl1 dimer binds to the PH domain of Akt/PKB forming large Tcl1-Akt/PKB complexes of unknown stoichiometry. Then, Akt/PKB is phosphorylated by PDK (3-phosphoinositide-dependent protein kinase 1) at Thr308 and at Ser473 by an unknown kinase. Once activated, Akt/PKB phosphorylates its downstream targets in the cytoplasm and possibly in the nucleus.

Our analysis of Tcl1 proteins has suggested another possible type of interaction with Akt/PKB. The consensus sequence for Akt/PKB phosphorylation is reported to be RxR-x(2)-[ST][22]. Human Tcl1, Mtcp1, and mouse Tcl1 show only one conserved and solvent accessible serine (Ser89 in human Tcl1) in the turn between beta strands 6 and 7 (Fig. 1 and 3). This conserved serine is also present in human Tcl1b. The sequence around the conserved serine has the pattern of RxR-x(3)-S in human and mouse Tcl1 and RxM-x(3)-S in Mtcp1 (Fig. 1). The sequence in Tcl1 resembles the consensus pattern for Akt/PKB phosphorylation, although there is an extra residue between the conserved RxR and S. However, the sequence consensus of a phosphorylation site can be degenerate. For example, a tyrosine kinase can phosphorylate sequences of different consensus patterns such as [RK]-x(2)-[DE]-x(3)-Y or [RK]-x(3)-[DE]-x(2)-Y (23;24). Therefore, it is possible that Akt/PKB can phosphorylate the conserved serine in the Tcl1 proteins. Alternately, the Rx[RM]-x(3)-S region may act as a substrate analog of Akt/PKB. The importance of the conserved Ser89 in human Tcl1 is consistent with its location being on the Akt/PKB interacting surface about 16 and 11Å from the residues Asp16 and Ile74, respectively (Fig. 3D).

Because Akt/PKB is important for proliferation and survival of lymphocytes, abnormal coactivation by Tcl1 in mature lymphocytes can lead to malignancies. Differences in the oligomeric form, subunit interface, surface accessible residues, and potential phosphorylation sites between Tcl1 and Mtcp1 proteins are likely to contribute to the tenfold differences[5,6] in their interactions with Akt/PKB. Hence, despite the highly conserved tertiary structures of the Tcl1 proteins, each Tcl1 family member may interact with different cellular proteins. These interacting proteins may be different isoforms of Akt/PKB or other unidentified proteins. Presently, only a few of the Tcl1 residues important for interactions with Akt/PKB have been experimentally defined. Further analysis is needed to fully define the interacting residues in the Tcl1-Akt/PKB complexes as well as the stoichiometry of such complexes. This review provides a rationale for more detailed mutational analysis of the Tcl1 proteins.

## ACKNOWLEDGEMENTS

This work was supported by the National Cancer Institute grant CA76259. J.M.P. was supported in part by the Georgia State University Research Program Enhancement grant.
